# Effects of β-Mannanase and Bacteriophage Supplementation on Health and Growth Performance of Holstein Calves

**DOI:** 10.3390/ani11020372

**Published:** 2021-02-02

**Authors:** Sinyong Jeong, Namchul Jo, Jung-Jin Lee, Jae-Hwan Lee, Dong-Keun Kam, Jakyeom Seo, Ermias Kebreab, Seongwon Seo

**Affiliations:** 1 Division of Animal and Dairy Sciences, Chungnam National University, Daejeon 34143, Korea; syjeong89@gmail.com (S.J.); jonamchul84@gmail.com (N.J.); jseo81@pusan.ac.kr (J.S.); 2 CTCBIO, Inc., Seoul 05724, Korea; jjlee@ctcbio.com (J.-J.L.); johneylee@ctcbio.com (J.-H.L.); 3 Cargill Agri Purina, Inc., Seongnam, Gyeonggi 13630, Korea; Dongkeun_Kam@cargill.com; 4 Department of Animal Science, University of California, Davis, CA 95616, USA; ekebreab@ucdavis.edu

**Keywords:** β-mannanase, enzyme, bacteriophage, feed additives, calf, growth, survival

## Abstract

**Simple Summary:**

For sustainable animal agriculture, we need to find ways to increase growth efficiency without using feed antibiotics. Bacteriophages, which are only harmful to specific bacterial strains, have been suggested as a feed additive replacing antibiotics. β-mannanase, which degrades mannan, is known to promote nutrient digestibility, animal growth, or both, thus improving feed efficiency. The objective of this study was to evaluate the effects of dietary supplementation with bacteriophage and β-mannanase on health and growth performance in calves. We assigned 36 pre-weaning male Holstein calves to one of four treatments with a 2 × 2 factorial arrangement: no supplementation, 0.1% β-mannanase, 0.1% bacteriophage, and both 0.1% bacteriophage and 0.1% β-mannanase supplementation in a starter on a dry matter basis. Compared to unsupplemented, the bacteriophage supplemented group showed a tendency to improve the survival rate without growth promotion. Supplementation of β-mannanase, on the other hand, increased the starter intake and the weekly body weight (BW) gain and tended to increase the final BW. Our study indicated that bacteriophage supplementation has a positive effect on survival rate, while β-mannanase supplementation improves growth performance in calves.

**Abstract:**

The objective of this study was to evaluate the effects of dietary supplementation with bacteriophage and β-mannanase on health and growth performance in calves. Thirty-six pre-weaning male Holstein calves were randomly allocated to one of four dietary treatments with a 2 × 2 factorial arrangement: no supplementation, 0.1% β-mannanase, 0.1% bacteriophage, and both 0.1% bacteriophage and 0.1% β-mannanase supplementation in a starter on a dry matter basis. The experiment lasted from 2 weeks before weaning to 8 weeks after weaning. Twenty-two calves survived to the end of the experiment. No interaction was observed between the two different feed additives. The bacteriophage supplementation tended to increase the odds ratio of survival (*p* = 0.09). The number of *Escherichia coli* in feces significantly decreased by bacteriophage supplementation one week after weaning. β-mannanase supplementation increased the concentrate intake (*p* < 0.01) and tended to increase the final BW (*p* = 0.08). Analysis of repeated measures indicated β-mannanase supplementation increased weekly body weight gain (*p* = 0.018). We conclude that bacteriophage supplementation may have a positive effect on calf survival rate, while β-mannanase supplementation may increase the growth rate and starter intake by calves just before and after weaning.

## 1. Introduction

Diarrhea and respiratory disease are the main causes of death in calves [1] and are caused by infection with viral, protozoal, and bacterial pathogens [2,3]. Calf mortality rates are higher in winter than in summer [4], mainly due to cold temperatures and poor ventilation [5]. Supplementation with antibiotics helps to prevent disease and promote growth in livestock [6]. However, several countries have restricted the use of antibiotics in the diets of livestock [7], and some others are in the process of banning antibiotics due to concern for antimicrobial resistance.

Several studies have been conducted to develop feed additives to replace the use of antibiotics as feed additives. For example, bacteriophages can repress the growth of specific or narrow groups of bacteria [8]. Bacteriophages multiply in bacteria by exploiting the biosynthetic machinery of the host [8]. Bacteriophages have shown the potential for various applications for human and animal health [8,9]. The bacteriophage supplementation in the diets of monogastric animals has been reported to reduce pathogenic bacteria in the intestine [10,11]. However, few studies have investigated the use of bacteriophages as a feed additive for ruminants. Prebiotics, such as mannan oligosaccharides (MOS), can also be added to the diet to enhance the health-improving activities of gut microbes [12,13]. The MOS can be produced by hydrolysis of mannan in feed by supplementation of β-mannanase, which can indirectly provide MOS to animals. Supplementation of β-mannanase in a diet improved feed utilization and animal performance in chicken [14,15], pigs [16,17], and ruminants [18,19,20]. Both supplementations are expected to improve gut health but via different modes of action.

Thus, the objective of the present study was to investigate the effects of bacteriophage and β-mannanase supplementation and their synergies, if any, on health, growth performance, intestinal pathogenic bacteria, and survival rate of calves reared in winter.

## 2. Materials and Methods

This study was conducted at the Center for Animal Science Research, Chungnam National University, Korea. Animal use and experimental protocols were reviewed and pre-approved by the Chungnam National University Animal Research Ethics Committee (approval number CNU-00188).

### 2.1. Animals and Housing

A total of 36 Holstein male calves (45.1 ± 7.30 kg of body weight (BW), averaged 3 to 4 weeks old) were purchased from several commercial dairy farms and used in this study. The calves were housed in individual wood pens (1.2 m width × 1.4 m length × 1.2 m height) at the Center for Animal Science Research, Chungnam National University. Pens were designed to prevent physical contact between calves but allow visual and auditory contact. The front of each pen had two openings for access to pails (diameter = 34 cm, height = 29 cm) mounted on the outside. The first seven days were used to adapt the calves to the diet by providing them free access to feed and water in individual indoor pens bedded with straw. During the experimental period, the pens were bedded with copra meal pellets. The bedding was replaced every 2 weeks throughout the experimental period.

### 2.2. Experimental Design and Diets

The experiment was carried out with 1 week adaptation and 10 week experimental periods. The experimental periods included 2 weeks prior to weaning and 8 weeks after weaning. Calves were divided into four groups and used in a balanced and completely randomized design with a 2 × 2 factorial arrangement. The dietary treatments were as follows: (1) control diet with neither supplementation (CON), (2) no bacteriophage but 0.1% β-mannanase (EZ), (3) 0.1% bacteriophage but no β-mannanase (BP), and (4) both 0.1% bacteriophage and 0.1% β-mannanase supplementation (BP_EZ) in a calf starter on a dry matter basis. The bacteriophage and β-mannanase used in this experiment were commercial feed additive BacterPhage C (CTCBIO, Inc., Seoul, Korea) and CTCZYME (CTCBIO, Inc., Seoul, Korea), respectively. BacterPhage C was composed of various bacteriophages targeting *Salmonella typhimurium*, *Salmonella enteritidis*, *Salmonella dublin*, *Salmonella derby*, *Staphylococcus aureus*, *Escherichia coli* k99, and f41, and *Clostridium perfringens* type A and C. CTCZYME contains Endo 1-4 β-mannanase (800,000 U/kg), which is produced using a patented strain of *Bacillus subtills* WL-7 (patent 100477456; CTCBIO, Inc., Seoul, Korea).

During the adaptation period, the calves were fed only a commercial milk replacer (Telilac; LNB International Feed B. V., Nisterlrode, The Netherlands; 21% crude protein (CP), 16% crude fat (CF)). The milk replacer was prepared with 125 g/L as the manufacturer’s recommendation and given to the calves at 10% BW. After a 1 week of adaptation, calf starter and timothy hay (Table 1) were offered for ad libitum intake before weaning, and thus, the feeding of the experimental diets started 2 weeks before weaning. The amount of milk replacer was progressively reduced by 20% daily from five days before weaning. From 1 week after weaning, the amount of starter was restricted to 1.5% body weight (BW) to prevent digestive disorders and enhance forage intake. The calves were fed twice daily at 0800 h and 1800 h with equal amounts of each meal throughout the experimental period. Drinking water was freely accessible to calves throughout the experimental period. Individual daily dry matter intake (DMI) was recorded by measuring the feed offered, and the feed refused. Body weight was measured before morning feeding at the start of the adaptation period, 2 weeks before weaning, at weaning, and weekly thereafter.

### 2.3. Enumeration of Intestinal Pathogenic Bacteria

Approximately 100 g of fecal samples were collected directly from the rectum following BW measurements 2 weeks before weaning, at weaning, and weekly thereafter. Collected fecal samples were placed on ice, transferred to the laboratory, and stored at −80 °C until analysis. One-gram of feces was diluted in 9 mL of phosphate-buffered saline (PBS) and homogenized by vortexing. Serial dilutions in PBS were plated in triplicate on the following selective media: eosin methylene blue agar (EMB agar, Oxoid Ltd., Basingstoke, UK), Salmonella Shigella agar (SS agar, Difco Laboratories, Detroit, MI, USA), and Perfringens agar (OPSP agar, Oxoid Ltd., Basingstoke, UK), which were used to isolate *E. coli*, *Salmonella* spp., and *C. perfringens*, respectively. All plates were incubated in an anaerobic chamber (Coy Laboratory Products Inc., Ann Arbor, MI, USA). *E. coli* and *Salmonella* spp. were incubated for 24 h at 37 °C, and *C*. *perfringens* was incubated for 18–24 h at 35 °C. On the EMB agar, colonies with a metallic green sheen were suspected to be *E. coli* and were counted. Colonies of *Salmonella* spp. on the SS agar had black centers without color and were counted. *Clostridium perfringens* produces black colonies on Perfringens agar.

### 2.4. Hematological Parameters

Before the beginning of the experiment, blood samples were taken from the jugular veins of all calves into a Vacutainer tube containing EDTA (Becton Dickinson Vacutainer Systems, Plymouth, UK). Approximately 2 mL blood samples were analyzed for initial immunoglobulin G (IgG) using Bovine IgG ELISA Core Kit (Koma Biotech, Seoul, Korea). After that, blood samples were taken from the jugular veins of 16 calves, which included four calves randomly selected from each treatment group. A total of six samples were taken from each calf throughout the experiment: 2 weeks before weaning, at weaning, and 1, 2, 5, and 8 weeks after weaning. Approximately 10 mL blood samples were taken from the jugular vein and collected into a Vacutainer tube containing EDTA (Becton Dickinson Vacutainer Systems, Plymouth, UK), as well as a serum tube containing clot activator (Becton Dickinson Vacutainer Systems, Plymouth, UK) before morning feeding. The EDTA tubes were placed on ice and then immediately transferred to the analytical laboratory of the Animal Hospital at Chungnam National University for complete blood count (CBC) analysis, which included white blood cells (WBC), lymphocytes, monocytes, neutrophils, eosinophils, basophils, the neutrophil to lymphocyte ratio (N:L), red blood cell (RBC), mean cell volume (MCV), hematocrit (HCT), mean cell hemoglobin (MCH), mean corpuscular hemoglobin concentration (MCHC), hemoglobin (Hgb), red blood cell distribution width (RDW), platelets (PLT), and mean platelet volume (MPV). Serum was obtained by centrifugation at 1300× *g* for 15 min at 4 °C and frozen at −80 °C until later analysis. The serum was analyzed for total protein, blood urea nitrogen (BUN), and glucose using kits purchased from Wako Pure Chemical Industries, Ltd. (Osaka, Japan) and a clinical auto analyzer (Toshiba Acute Biochemical Analyzer-TBA-40FR, Toshiba Medical instruments, Tokyo, Japan) following the procedures described by Wang et al. [21].

### 2.5. Chemical Analysis

Feed samples were sampled one before the beginning of the experiments and were ground through a cyclone mill (Foss, Hillerød, Denmark), fitted with a 1 mm screen prior to chemical analysis. Contents of dry matter (DM #934.01), crude protein (CP; #976.05), ether extract (EE; #920.39), acid detergent fiber (ADF; #973.18), and ash (#942.05) were determined as previously described AOAC (Association of Official Analytical Chemists) [22]. CP was calculated as 6.25 times the nitrogen content, and total nitrogen was measured by the Kjeldahl method using a DK 20 Heating Digester and Semi-Automatic Distillation Unit Model UDK 139 (VELP Scientifica, Usmate, Italy). Neutral detergent fiber (NDF) was analyzed using a heat stable amylase and expressed inclusive of residual ash (aNDF) as described by Van Soest et al. [23].

### 2.6. Statistical Analysis

Data were analyzed using the MIXED procedure in SAS (SAS Institute Inc., Cary, NC, USA) for comparison of means. The linear model was as follows

Y_ij_ = *μ* + *τ*_i_ + *e*_ij_

where Y_ij_ is jth observation (j = 1–12) in *i*th treatment (i = 1–4), *μ* is the overall mean, *τ*_i_ is the fixed effect of the *i*th treatment, and *e*_ij_ is the unexplained random effect on the *j*th observation in the *i*th treatment.

Three orthogonal contrasts were tested: the difference with or without bacteriophage supplementation (i.e., CON and EZ versus BP and BP_EZ), the difference with or without β-mannanase supplementation (i.e., CON and BP versus EZ and BP_EZ), and the interaction between bacteriophage and β-mannanase supplementation (i.e., CON and BP_EZ versus EZ and BP). When no interaction was observed, we further tested the main effect of the two supplementations orthogonally without considering the interaction between them [24].

The effects of treatments on BW and hematological parameters were analyzed using the MIXED procedure with repeated measures. Initial values were included in the model as covariates in each analysis. The best variance-covariance structures for each analysis were unstructured, UN, and auto-regression, AR(1), for BW and hematological parameters, respectively, as assessed by the lowest Akaike information criterion (AIC) and Bayesian information criterion (BIC). In the BW analysis, the treatment × week interaction, body weight gain (BWG, kg/week), was of particular interest. To estimate the effects of bacteriophage and β-mannanase supplementation on survival rate, we used the logistic procedure in SAS to estimate the strength of the association between each variable and death based on the odds. Pair-wise comparisons of the least square means were conducted using the PDIFF option with Tukey–Kramer adjustment when there was a significant overall treatment effect. Significance was declared at *p* < 0.05, and a trend was discussed at 0.05 ≤ *p* < 0.10.

## 3. Results

### 3.1. Effects of Supplementation of Bacteriophage

**Figure 1:**
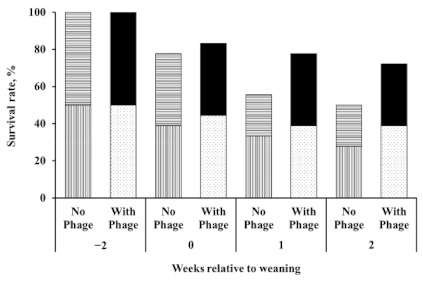
Calf survival rate. Striped bars and dotted or solid bars represent the survival rate in the bacteriophage unsupplemented group (i.e., control and β-mannanase) and the bacteriophage supplemented group (i.e., bacteriophage and bacteriophage plus β-mannanase supplementation), respectively. No further calf deaths occurred from two weeks after weaning.

Twenty-two calves survived to the end of the experiment. Among the treatments, five, four, seven, and six calves survived in the CON, EZ, BP, and BP_EZ groups, respectively. No calves died later than 2 weeks post-weaning. Although not statistically significant (*p* = 0.21), the bacteriophage supplemented group (BP and BP_EZ) showed a numerical increase in calf survival rate, which was defined as the number of surviving calves as a percentage of the total number of calves at the beginning of the experiment, compared to the bacteriophage unsupplemented group (CON and EZ). After weaning, the survival rate rapidly declined in the bacteriophage unsupplemented group, compared to that in the bacteriophage supplemented group (Figure 1). Up to 2 weeks after weaning, 50% of the calves in the bacteriophage unsupplemented group survived, while 70% of the calves in the bacteriophage supplemented group survived.

Differences in the odds ratio of survival between the bacteriophage unsupplemented and supplemented groups were also compared using the logistic procedure with initial BW as a covariate. Initial BW significantly affected the odds ratio of survival. The estimated effect of initial BW on the odds ratio of survival was 1.41 (*p* < 0.01). Therefore, a 1-kg increase in BW 2 weeks before weaning resulted in a 1.41-fold increase in survival. Accounting for differences in the initial BW of the calves, the odds ratio of survival tended to increase approximately 6.15-fold in the bacteriophage supplemented group (*p* = 0.09).

The supplemented bacteriophage did not show a difference in growth performance except for the tendency to decrease the forage DMI (*p* = 0.07) (Table 2).

### 3.2. Effects of Supplementation of β-Mannanase

No significant differences were observed among treatments for the initial BW, BW at weaning, and final BW of calves (Table 2). In contrast, comparisons showed that the calves supplemented with β-mannanase tended to have a heavier final BW (*p* = 0.08) than those unsupplemented with β-mannanase. Total DMI did not differ among the treatment groups (*p* > 0.10); however, the DMI of concentrate was significantly increased by β-mannanase supplementation (*p* < 0.01). No significant differences were observed among treatments for feed efficiency or the average daily gain (g) to DMI (g) ratio (*p* > 0.05).

**Figure 2:**
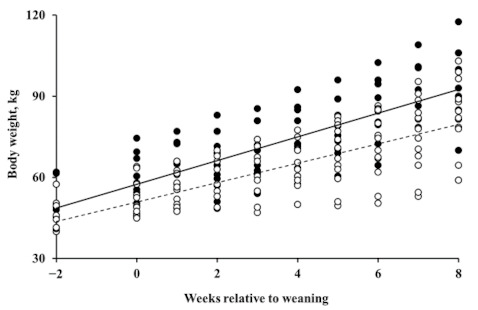
Body weight (BW) of calves at weeks relative to weaning. The open circle (○) and dotted line represent BW of the β-mannanase unsupplemented group (i.e., control and bacteriophage supplementation) and the closed circle (●) and solid line represent BW of the β-mannanase supplemented group (i.e., β-mannanase and bacteriophage plus β-mannanase supplementation).

The effects of dietary treatments on changes in BW were analyzed using the 22 surviving calves. In contrast to the health of the calves, no trend was observed for the effect of bacteriophage supplementation on growth by orthogonal contrasts analysis; however, BWG was significantly greater in the β-mannanase supplemented group (*p* = 0.018). The estimated BWG was 3.05 and 4.09 kg/week, equivalent to 436 and 584 g/d, in the β-mannanase unsupplemented and supplemented groups, respectively (Figure 2).

### 3.3. Intestinal Pathogenic Bacteria and Hematological Parameters

**Figure 3:**
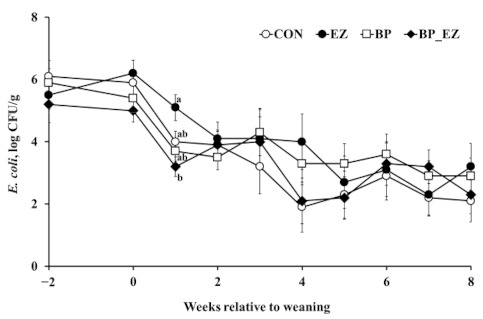
Changes in the number (log CFU/g) of *Escherichia coli* in calf feces. The open-circle (○), closed-circle (●), open-square (□), and closed-diamond (♦) represent least square means of the control, β-mannanase supplementation, bacteriophage supplementation, and bacteriophage plus β-mannanase supplementation groups, respectively. Error bars represent standard errors of the means at each week. ^a,b^ Means that do not have common superscripts differ (*p* < 0.01); CFU: colony forming unit.

The total number of fecal *E. coli* decreased linearly throughout the experimental period (*p* < 0.01, Figure 3). Among treatments, there was a significant difference in the number of *E. coli* 1 week after weaning (*p* = 0.01). After 1 week weaning, the bacteriophage supplemented group had a significantly reduced population of *E. coli* in feces compared with the bacteriophage unsupplemented group (3.5 versus 4.7 log colony forming unit [CFU]/g, *p* < 0.01). Thereafter, no difference between groups was found. *Salmonella* spp. and *C. perfringens* were barely detectable in fecal samples from calves throughout the experimental period (data not shown).

Most of the tested hematological parameters were not significantly different among the treatment groups; however, the lymphocyte concentration and the neutrophil to lymphocyte (N:L) ratio tended to differ by treatment (*p* = 0.07 and 0.08, respectively; Table 3). This was due to the interaction between β-mannanase and bacteriophage supplementation. Unlike supplementation of β-mannanase alone to control (CON vs. EZ), supplementation of β-mannanase in addition to bacteriophage (BP vs. BP_EZ) decreased the concentration of lymphocytes in blood (*p* < 0.01). There was a significant interaction between β-mannanase and bacteriophage supplementations (*p* = 0.02). When only β-mannanase was supplemented to control (CON vs. EZ), the N:L ratio substantially increased. However, this increase was lowered when β-mannanase was co-supplemented with bacteriophage (BP vs. BP_EZ).

## 4. Discussion

The most important causes of death in the early life of cattle are diarrhea and respiratory disease, resulting in significant economic losses to the cattle industry [1]. Many substances (e.g., antibiotics, probiotics, and prebiotics) have been proposed to reduce the calf mortality rate through direct antimicrobial effects or through improved animal health. In line with these efforts, this study investigated the effects of dietary supplementation with a commercial bacteriophage product, a commercial β-mannanase, and their associative effects on health and growth performance in male Holstein calves. This is the first study to evaluate the effect of bacteriophage supplementation and its interaction with β-mannanase supplementation in ruminants.

By the end of the experiment, 14 out of 36 calves had died up to 2 weeks after weaning, and the overall calf mortality rate was 39%. The major cause of death was infection by coronavirus and coccidiosis, as diagnosed by the Animal Disease Diagnostic Division of the Ministry for Food, Agriculture, Forestry and Fisheries (Anyang, Gyeonggi, Korea). Although no national statistics on calf mortality are available in Korea, this value was higher than expected. Mortality rates in the early life of cattle can vary widely by season, region, and country [1]. Reported values vary from 1.5% [25] to 25% [26]. Even in developed countries, the mortality rate could be up to 31% [27].

One possible reason for the high calf mortality rate is the inadequate passive transfer of immunoglobulins as a result of the insufficient consumption of colostrum. Calf immunity depends on the passive transfer of colostrum immunoglobulins from dams. However, failure of the passive transfer of immunoglobulins was reported in a considerable proportion of dairy calves [28], which occurred even when the calves remained with their dam after birth [29]. Insufficient passive transfer of immunoglobulins is associated with increases in morbidity and mortality before and after weaning [28]. At the time when this trial was conducted, the price of male Holstein calves was so low that many dairy farmers paid little attention to the supply of adequate amounts of colostrum to male calves. The mean (± standard deviation (SD)) serum IgG concentration of the calves, measured by ELISA on the day the experiment, was initiated was 10.8 (±3.34 g/L). This implied that the passive transfer of IgG was inadequate (<16 g/L; [30]), probably due to insufficient consumption of colostrum after birth.

The cold weather may be another reason for the high calf mortality rate. The experiment was conducted between January and March in unusually cold winter. The weekly mean average (minimum) daily temperature during the 2 weeks before weaning, 1 week before weaning, 1 week after weaning, and 2 weeks after weaning were −2.2 °C (−5.8), 1.9 °C (−2.7), −4.1 °C (−8.5), and −0.1 °C (−4.8), respectively. To maintain the indoor temperature, ventilation was relatively poor. Calf mortality rate is higher in winter than in summer [1,4], and poor ventilation is known to increase the calf mortality rate during cold winters [5]. Therefore, we cannot exclude the possibility that the positive effects of the supplementations observed in this study can be limited to harsh conditions (e.g., failure of passive immunity transfer and inadequate environmental conditions).

Although statistical significance was not attained on survival rate among the treatments, bacteriophage supplementation tended to increase the odds ratio of survival of calves when the effect of initial BW was considered as a covariate. Supplementation of diets with bacteriophage also suppressed the population of *E. coli* (3.5 versus 4.7 log CFU/g) at 1 week after weaning, when four calves died in the bacteriophage unsupplemented group, and one calf died in the bacteriophage supplemented group. Studies have shown that the population of *E. coli*, *Salmonella* spp., and *C. perfringens* in feces is associated with the survival rate of young ruminants [31,32].

The use of bacteriophages as bacteria-control agents has been proposed in several studies [33,34]. Inoculation of phage in sheep decreased populations of *E. coli* O157:H7 in the rumen, cecum, and rectum [35]. When challenged with pathogens, the application of pathogen-specific bacteriophages decreased the number of intestinal pathogens and reduced mortality in chickens [36]. Supplementation of commercial feed additive with bacteriophages has reduced fecal *E. coli* counts in laying hens [37], broilers [38], and pigs [39]. However, increased *E. coli* counts following supplementation have also been reported in laying hens [10].

Although supplementation with bacteriophages may improve the health status of calves, it may not increase their growth performance. In the present study, the growth of calves was not affected by bacteriophage supplementation. Inconsistent effects of bacteriophage supplementation have been reported on the growth of farm animals. In pigs, DMI, average daily gain (ADG), and feed efficiency were not improved by bacteriophage supplementation at 0.5 g/kg although apparent total tract digestibility (ATTD) of DM and nitrogen were increased [39]. On the other hand, Kim et al. [11] and Hosseindoust et al. [40] reported that 1.0 and 1.5 g/kg bacteriophage had significantly improved ADG and the fecal score of pigs. Bacteriophage supplementation did not improve the performance of chickens. No significant differences in growth and ATTD of nutrients were observed in broilers [38]. Little improvement in laying performance and egg quality was observed following supplementation in laying hens [10].

Co-supplementation of β-mannanase and bacteriophage seemed to alter the inflammatory response of calves, although changes in hematological parameters are hard to interpret due to large variations among individual animals. When both bacteriophage and β-mannanase supplementation were provided, a reduction in the lymphocyte count without an increase in the neutrophil count was observed. This may imply that chronic inflammation was reduced, although the mode of action is not clear. Chronic viral infections, inflammation, and autoimmune diseases increase the numbers of both neutrophils and lymphocytes [41]. Both neutrophil and lymphocyte counts were also increased in vaccinated animals [42]. Although temporary decreases in the number of lymphocytes can occur [43], this may not be the case in the present study since the lymphocyte counts were measured six times throughout the 10 week experimental period, and a concurrent decrease in the number of neutrophils was not observed. Stress may also cause lymphocyte counts to decrease; however, under stressed situations, the number of neutrophils is generally increased [44]. Other studies reported no differences in hematological parameters following bacterial supplementation in pigs [39] and broilers [38].

Conversely, β-mannanase supplementation increased BWG (*p* = 0.018). Numerous studies in monogastric animals have reported that β-mannanase supplementation improved feed utilization and growth performance in broilers [14,45] and pigs [16,46]. Positive effects of β-mannanase supplementation on growth performance have also been reported in ruminants. The inclusion of β-mannanase in the starter feed tended to improve the feed efficiency of calves [20]. In addition, β-mannanase supplementation improved ADG, feed efficiency, and nitrogen retention in growing goats [18]. In dairy cows, the supplementation of β-mannanase has increased the feed efficiency by lowering the somatic cell count [19].

The mechanism of growth improvement in response to β-mannanase supplementation in ruminants remains to be determined. In monogastric animals, β-mannanase supplementation has been proposed to improve growth performance by improving nutrient digestibility and utilization in broilers and in pigs [16,47]. Another proposed mechanism of action of β-mannanase supplementation is associated with mannan oligosaccharides, which are released following the breakdown of β-mannan in the diet and may stimulate the innate immune system of animals [14]. However, in ruminants, Lee et al. [18] observed a decrease in nutrients digestibility and proposed that increased growth following β-mannanase supplementation might be associated with improved nitrogen metabolism in goats. Furthermore, β-mannanase supplementation alone did not alter the innate immune system in the present study. Further studies are needed to obtain a better understanding of the mechanism of action of β-mannanase supplementation in ruminants.

## 5. Conclusions

Supplementation of feed with bacteriophage may have a positive effect on calf survival rate but not on growth performance. The levels of some pathogenic bacteria (e.g., *E. coli*) may be reduced following bacteriophage supplementation. β-mannanase supplementation, on the other hand, may increase the growth rate and intake of a starter feed in calves. No synergistic effects were observed between the two dietary supplements on intake, growth performance, and mortality of calves, although a reduction in the lymphocyte count without an increase in the neutrophil count was observed when the diet was supplemented with both bacteriophage and β-mannanase.

## Figures and Tables

**Table 1:** Chemical composition of the experimental diets (g/kg DM or as stated).

	Experimental Diets					
Item ^1^	CON ^2^	EZ	BP	BP_EZ	Timothy	Milk Replacer
DM (g/kg as fed)	889	889	888	886	928	952
CP	216	219	200	226	60	211
EE	67	64	58	61	12	140
Ash	56	65	59	62	37	104
aNDF	308	327	362	377	713	-
ADF	223	245	276	293	470	-

^1^ DM: dry matter; CP: crude protein; EE: ether extract; aNDF: neutral detergent fiber analyzed using a heat-stable amylase and expressed inclusive of residual ash; ADF: acid detergent fiber. CON: control calf starter; EZ: control diet supplemented with 0.1% β-mannanase; BP: control diet supplemented with 0.1% bacteriophage; BP_EZ: control diet supplemented with 0.1% bacteriophage and 0.1% β-mannanase on a dry matter basis. ^2^ 195 g/kg corn, 150 g/kg wheat, 100 g/kg corn gluten meal, 80 g/kg soy hulls, 80 g/kg cottonseed hulls, 50 g/kg soybean meal, 50 g/kg rapeseed meal, 50 g/kg palm kernel meal, 50 g/kg rice bran, 50 g/kg gluten feed, 50 g/kg DDGS, 40 g/kg molasses, 20 g/kg copra meal, 20 g/kg animal fat, 15 g/kg limestone on an as-fed basis.

**Table 2:** The effects of bacteriophage and β-mannanase supplementation on feed intake and growth performance in male Holstein calves.

							*p*-Value			
		Treatment ^1^						Contrast		
Item ^2^	n	CON	EZ	BP	BP_EZ	SEM ^3^	Overall	EZ	BP	Interaction
BW, kg										
Initial BW	36	44	47	44	45	2.7	0.84	0.40	0.77	0.80
BW at weaning	29	52	55	50	56	3.4	0.56	0.18	0.81	0.61
Final BW	22	87	99	77	89	7.3	0.16	0.08	0.17	0.99
ADG, g	22	569	638	451	587	73.7	0.21	0.13	0.21	0.61
Intake, g/d										
DMI	22	1612	1834	1402	1624	149.6	0.18	0.11	0.14	1.00
Concentrate DMI	22	802	993	759	921	71.0	0.06	0.01	0.46	0.82
Forage DMI	22	700	715	538	590	92.2	0.34	0.68	0.07	0.82
Feed Efficiency ^4^	22	0.36	0.32	0.35	0.36	0.032	0.60	0.40	0.59	0.30

^1^ CON: control calf starter; EZ: control diet supplemented with 1 g/kg β-mannanase; BP: control diet supplemented with 1 g/kg bacteriophage; BP_EZ: control diet supplemented with 1 g/kg bacteriophage and 1 g/kg β-mannanase. ^2^ BW: body weight, ADG: average daily gain, DMI: dry matter intake. ^3^ SEM: standard error of means. ^4^ ADG (g)/DMI (g).

**Table 3:** The effects of bacteriophage and β-mannanase supplementation on hematological responses in Holstein calves (*n* = 4 per treatment). Means of five samples are presented (at weaning and at 1, 2, 5, and 8 weeks after weaning).

						*p*-Value			
	Treatments ^1^						Contrast		
Item ^2^	CON	EZ	BP	BP_EZ	SEM ^3^	Overall	EZ	BP	Interaction
Leukocytes									
WBC, 10^3^/μL	7.6	8.7	7.7	9.2	0.53	0.66	0.75	0.76	0.24
Lymphocytes, 10^3^/μL	3.2	3.1	3.2	2.9	0.16	0.07	0.66	0.94	<0.01
Monocytes, 10^3^/μL	0.75	0.93	0.74	0.76	0.111	0.28	0.22	0.26	0.29
Neutrophils, 10^3^/μL	2.3	2.2	2.3	2.4	0.12	0.18	0.68	0.71	0.03
Eosinophils, 10^3^/μL	0.44	0.47	0.46	0.57	0.068	0.27	0.19	0.64	0.16
Basophils, 10^3^/μL	0.02	0.03	0.03	0.02	0.004	0.15	0.40	0.04	0.56
N:L	0.81	0.93	0.81	0.91	0.13	0.08	0.48	0.46	0.02
Erythrocytes									
RBC, 10^6^/μL	7.1	7.3	7.5	6.8	0.31	0.53	0.66	0.31	0.35
MCV, fL	35.6	34.0	35.5	34.2	0.71	0.23	0.26	0.56	0.10
HCT, %	25.1	25.3	26.3	23.4	1.16	0.58	0.96	0.45	0.25
MCH, pg	12.0	11.8	12.0	11.9	0.16	0.85	0.49	0.57	1.00
MCHC, g/dL	34.2	34.8	34.5	35.7	0.51	0.78	0.88	0.72	0.34
Hgb, g/dL	9.3	8.5	8.8	8.2	0.59	0.41	0.30	0.59	0.22
RDW, %	29.0	19.3	20.5	21.3	4.58	1.00	0.87	0.98	0.94
Thrombocytes									
PLT, 10^6^/μL	2.3	2.5	2.5	2.4	0.24	0.61	0.41	0.36	0.61
MPV, fl	16.2	16.8	16.7	16.7	0.37	0.67	0.83	0.48	0.33
Total protein, mg/dL	6.1	5.9	6.1	6.3	0.18	0.84	0.47	0.85	0.62
BUN, mg/dL	11.5	9.5	12.0	12.1	0.70	0.56	0.65	0.29	0.40
Glucose, mg/dL	68.7	74.3	64.1	63.1	3.51	0.72	0.29	0.63	0.99

^1^ CON: control calf starter; EZ: control diet supplemented with 1 g/kg β-mannanase; BP: control diet supplemented 1 g/kg bacteriophage; BP_EZ: control diet supplemented with 1 g/kg bacteriophage and 1 g/kg β-mannanase. ^2^ WBC: white blood cell; N:L: neutrophil to lymphocyte ratio; RBC: red blood cell; MCV: mean cell volume; HCT: hematocrit; MCH: mean cell hemoglobin; MCHC: mean corpuscular hemoglobin concentration; Hgb: hemoglobin; RDW: red blood cell distribution width; PLT: platelets; MPV: mean platelet volume; BUN: blood urea nitrogen. ^3^ SEM: standard error of means.
